# Immunomodulatory Drugs in Melanoma Brain Metastases

**DOI:** 10.15190/d.2019.6

**Published:** 2019-06-26

**Authors:** Gil Nuno Castro Fernandes

**Affiliations:** Comenius University, Sasinkova 4, 813 72 Bratislava, Slovakia

**Keywords:** Immunotherapy, Immune Checkpoint Inhibitors, PD-1, PD-L1, CTLA-4; BRAF inhibitor; melanoma; brain metastases.

## Abstract

Brain metastases are about ten times more frequent than a brain primary tumor, being present in 20-40% of adults with systemic cancer. Together with lung cancer and breast cancer, skin cancers such as melanoma are top primary tumors which metastasizes to the brain. Advanced melanoma is well known for its propensity to metastasize to the brain, with 80% of patients presenting brain metastasis at the autopsy. However, current therapies are not very efficient and brain metastases are in most of the cases lethal. Treatment of melanoma brain metastases with surgery and/or radiation therapy results in a median overall survival of only about four months after diagnosis. New immunotherapies such as targeted or immunomodulatory drugs, many in clinical trials, have shown promise, with some immunomodulatory drugs being able to at least double the overall survival rates for patients with melanoma brain metastases. This review focuses on the recent advances and future potential of using immunotherapy, such as the newly developed immunomodulatory drugs, for melanoma brain metastases therapy. Immunomodulatory drugs bring a great promise as new tools for melanoma treatment in particular and for the treatment of other types of malignancies in general.

## SUMMARY

1. Introduction

2. Melanoma Brain Metastasis

3. Current Treatments in Melanoma Brain Metastasis

4. Immunotherapy in Metastatic Melanoma Tumors

5. Challenges and Limitations

6. Conclusion

## 1. Introduction

Brain metastasis, the spread of a tumor from a primary neoplasm to the brain, is about 10 times more frequent than a primary brain tumor^1^. Noteworthy, 20-40% of cancer patients with systemic pathology have or will develop brain metastases^2,3^. Most common brain metastases have their primary tumor in the lung (~45%), breast (20%) and skin (e.g. melanoma, 10%)^4^. Brain metastases have a very poor prognosis, being characterized by a progressive Central Nervous System (CNS) damage and functional decline, significantly affected quality of life and shortened patient survival. Advanced melanoma is well known for its potential to metastasize to the brain. Approximately 80% of the melanoma patients present brain metastases at autopsy^5,6^.

However, current therapies are not very efficient and brain metastases are in most of the cases lethal. Treatment of melanoma brain metastases with surgery and/or radiation therapy results in a median overall survival of only about 4-6 months after diagnosis and they cause notable complications and morbidity (stroke, radiationinduced necrosis and cognitive defects)^7^. New immunotherapies such as targeted or immunomodulatory drugs, many in clinical trials, have shown promise, with some immunomodulatory drugs being able to at least double the overall survival rates for patients with melanoma brain metastases^8^. Immunotherapy uses components of the body's own immune system to fight against cancer. It works in several ways, for example by enhancing the capacity of the immune system to attack cancer cells or giving the immune system specific components artificially produced^9^. In particular, immunomodulators, antibodies stimulating T-cell function either by blocking or activating regulatory receptors, have shown to cause regression of several types of tumors and an exponential number of clinical trials are underway. Several immunomodulatory drugs/ checkpoint inhibitors are already approved by the US Food and Drug Administration (FDA) for the treatment of melanoma, non-small cell lung cancer, breast cancer, bladder cancer, kidney cancer, Hodgkin lymphoma^10,11^. Noteworthy, pembrolizumab was recently approved by the FDA for solid tumors with microsatellite instability-high (MSI-H) or mismatch repair-deficient^11^.

Here, we aim to review the most important advances and future potential of using immunotherapy, such as the newly developed immunomodulatory drugs, for melanoma brain metastases therapy.

## **2. Melanoma Brain Metastasis**

Melanoma brain metastases have been detected in about 45-60% of the patients, with 75%-80% presenting brain metastases at autopsy examinations. MRI is the gold standard for both diagnosis and monitoring of brain metastases^12^. Patients diagnosed with melanoma brain metastasis have an overall survival of only 4 to 6 months with standard available treatments, such as surgery and/or radiation therapy^13^. This is definitely not the desired outcome and sustain efforts are currently underway to develop better therapies.

The tumor microenvironment is an important factor influencing all steps of metastasis development, from metastasis formation to its progression and response to different therapies. In addition to the tumor cells, tumor microenvironment also contains other types of cells, such as fibroblasts, immune cells, pericytes and endothelial cells. The main features distinguishing the brain tissue from any other tissues are the presence of blood-brain barrier (BB) and unique resident cells (microglia, astrocytes and neurons)^14^. Recent results suggest that tumor cells from brain metastases can communicate with local astrocytes through gap junctions and program them to produce and secrete tumor-stimulating cytokines. These cytokines will then promote NF-kB-mediated survival and/or proliferation of cancer cells. Gap junctions can be successfully targeted^15^.

Interestingly, the report of a functional lymphatic vasculature along dural sinuses in mice caused a revision of the previous view of CNS as an immune privileged site^16,17^. Moreover, CNS-derived antigens can induce an immune response in cervical lymph nodes^18^, while some reports show that the BB can be affected in brain tumors resulting in significant accumulation of immune cells from outside CNS^19^. Noteworthy, previous studies reported that the brain metastases, unlike normal brain parenchyma and primary CNS tumors, have an immunoregulatory environment significantly infiltrated by lymphocytes. For example, over 99.1% of the analyzed brain metastases in a study shown the presence of the CD3+ lymphocytes, with over 55% having high density of tumor-infiltrating lymphocytes^20^. This is in contrast with the early-stage brain tumors, which, at least in part, were previously shown to have an immunosuppressive environment, with no presence of peripheral immune cells^21,22^. Taken into consideration these results, it makes sense to consider immunotherapy as a potentially promising tumor-targeting strategy in melanoma brain metastases. Recent clinical trials have confirmed that his hypothesis is correct.

## **3. Current Treatments in Melanoma Brain Metastasis**

Current therapies employed for brain metastases are generally inefficient, with very low median overall survival. They include whole brain radiation therapy (WBRT), surgery and stereotactic radiosurgery (SRS)^23^. WBRT is the standard treatment for metastatic brain tumors, with WBRT and surgical removal being used for multiple and/or large tumors and MRI-assisted SRI for smaller tumors. Tumor Treating Fields method is an additional option used in treating brain metastases^24-27^. Although successful, it may result in seizures and other CNS symptoms, such as insomnia or anxiety^27^.

Treatment of melanoma brain metastases with surgery and/or radiation therapy results in a median overall survival of only about 4-6 months after diagnosis and they cause notable complications and morbidity^7^ (**Table 1**). In general, SRS is preferred to WBRT in the treatment of melanoma brain metastasis^28^. Melanoma cells usually have a powerful DNA damage repair machinery, resulting in the need of delivery of larger fractions/doses of radiotherapy^29^. In contrast, chemotherapy has produced disappointing results in melanoma patients with brain metastases, and the results are similar to those obtained in melanoma treatment in general^30^.

**Table 1:** Current Treatments in Melanoma Brain Metastasis

Treatment	Most Important Side Effects
Whole-body radiotherapy	Radiation toxicity Headaches Nausea Vomiting Bone marrow suppression Skin reactions Fatigue
Stereotactic radiosurgery	Neurocognitive decline Brain swelling Fatigue Skin problems Local hair loss Nausea Vomiting Headaches
Surgical resection	Repeated surgical traumas Pain Fatigue Infections Organ dysfunction Appetite loss
Brachytherapy	Damage of brain tissue Brain swelling

More promising results were obtained with targeted treatments in patients presenting BRAF activating mutations. For example, vemurafenib, dabrafenib or dabrafenib in combination with trametinib are FDA approved for metastatic melanoma patients that show the BRAFV600 mutation^31,32^ (see **Table 2**, Targeted Therapies).

**Table 2:** FDA Approved Drugs for Melanoma Treatment ^2-5,8,20-36^ GM-CSF - granulocyte-macrophage colony-stimulating factor; IL-2 - Interleukin 2; cuSCC - cutaneous squamous cell carcinoma;

Treatment	Mechanism of action	Effectiveness	Side Effects
IMMUNOTHERAPY			
Talimogene Laherparepvec (T-VEC)	Virus-mediated GM-CSF production (genetically modified live oncolytic herpes virus)	16.3% decrease in tumor size versus 2.1% in patients treated with GM-CSF; however, it does not improve overall survival; no effect on metastatic melanoma (e.g. brain metastasis)	Chills Fever Nausea Flu-like symptoms
Aldesleukin	Cytokine, targets the IL-2/IL-2R pathway	Effective in patients with advanced melanoma	Rash, Diarrhea Chills, Nausea
Peginterferon Alfa-2b	Cytokine, targets the IFNAR1 pathway, antiangiogenesis, direct action on tumor growth, allows interferon to stay longer in the blood	Efficient in delaying or preventing relapse of melanoma; however, it has no benefit on overall survival	Flu-like symptoms Fever Chills Headache Nausea Vomiting
High-dose Interferon Alfa-2b	Cytokine, targets the IFNAR1/2 pathway, antiangiogenesis, direct action on tumor growth	Effective prevention of melanoma relapse from 0.98 years to 1.72 years, 46% of patients taking it have five year-survival compared to 37% for those who did not take; it is the only approved drug for late stage IIB or IIC melanoma	Acute flu-like symptoms Nausea Vomiting Loss of appetite Low white and red blood cell counts
Pembrolizumab	Checkpoint inhibitor, targets the PD-1/PD-L1 pathway	At a dose of 2mg/kg, 24% of patients had their tumor shrink for about 1.4 to 8.5 months; similar effect at a dose of 10mg/kg; included in the adjuvant (pre-surgical) setting	Fatigue Cough Nausea Severe itching, Rash Joint pain
Nivolumab	Checkpoint inhibitor, targets the PD-1/PD-L1 pathway	35% reduction on the risk of recurrence or death in patients with stage III melanoma; 45% reduction in the risk of disease progression in patients with stage IV melanoma	Rash Pneumonitis, Colitis, Hepatitis Muscle or joint pains Muscle weakness Headache, Dizziness
Ipilimumab	Checkpoint inhibitor, targets the CTLA-4 pathway	First-line therapy for subsets of patients with advanced melanoma	Fatigue Diarrhea Itching, Rash
Combined Nivolumab and Ipilimumab Regimen	Targets both PD-1/PD-L1 and CTLA-4 pathways	58% of patients had a three-year overall survival rate compared to 52% to those who only took nivolumab; However, the toxicity tripled with the combination	Rash, Itching Headache Vomiting Colitis, Diarrhea
TARGETED THERAPIES			
Vemurafenib	Kinase inhibitor, blocks activity of V600E-mutated form of BRAF (protein helping the growth of melanoma)	About 50% of patients had their tumor shrink compared to 5% who received DTIC; Progression free-survival was 13.6 months compared to 9.7 months for patients on standard chemotherapy; On clinical trials, 77% of patients were still alive compared to 64% of those taking DTIC	cuSCC (24%) Severe allergic reaction Skin rash Photosensitivity reaction Joint discomfort Uveitis
Trametinib	Kinase inhibitor, blocks the activity of V600E and V600K mutated form of BRAF (key protein helping the growth of melanoma)	On phase III of clinical trial, progression-free survival was 4.8 months compared to 1.5 months for patients on chemotherapy; 22% of patients had their tumor shrink compared to 8% for chemotherapy	Skin rash (87%) Heart failure Shortness of breath Cough Blindness Blurred vision High blood pressure
Dabrafenib	Kinase inhibitor, blocks activity of V600E-mutated form of BRAF (key protein helping the growth of melanoma)	Progression free-survival was 6.9 months compared to 2.7 months for patients on standard chemotherapy; on phase III clinical trial, 52% of patients had their tumor shrink compared to 7% who received dacarbazine	cuSCC Primary melanoma Serious fever Diabetes Hair loss
Combined Trametinib and Dabrafenib Regimen	Blocks some mutated forms of BRAF and MEK kinases (proteins helping the growth of melanoma)	On stage III, 53% decreased risk of disease recurrence or death; on stage IV, after 1 year of treatment, the overall survival was 73% compared to 64% in vemurafenib therapy; after 2 years, 51% of the patients were still alive compared to 38% taking vemurafenib alone; progression-free survival was 12.6 months versus 7.3 months	Skin rash Higher incidence for basal cell carcinoma Primary melanoma Serious fever Hemorrhage Heart failure Blood clots
Combined Encorafenib and Binimetinib	Blocks some mutated forms of BRAF and MEK kinases (proteins helping the growth of melanoma)	Progression-free survival was 14.9 months compared to 7.3 months for the patients treated with vemurafenib alone; only 5% of patients stopped the treatment due to adverse reactions	Fatigue Nausea Diarrhea Joint pain/swelling Abdominal pain
Combined Cobimetinib and Vemurafenib Regimen	Blocks activity of V600E-mutated form of BRAF and MEK kinases (proteins helping the growth of melanoma)	Progression-free survival was 12.3 months compared to 7.2 months for the patients treated with vemurafenib alone; 65% of patients were alive 17 months after the beginning of treatment compared to 50% of those taking vemurafenib alone	Diarrhea Photosensitivity reaction Nausea Vomiting Fever
CHEMOTHERAPY			
Dacarbazine	Anti-mitotic; anti-metastatic	5-20% of patients with stage IV melanoma had their tumor shrink or growing slowly; however, it doesn’t improve progression-free or overall survival; it’s the only approved drug to treat metastatic melanoma	Allergic reactions Blood problems Nausea, Vomiting Diarrhea Flu-like symptoms

However, although these targeted inhibitors are effective when used, resistance emerges in most of the treated cases. Resistance occurrence in melanoma brain metastases is not well studies and the specific CNS environment may contribute to different resistance mechanisms compared to the mechanisms already described in melanoma located outside the brain^35,36^.

In recent years, already FDA approved or in clinical trials immunotherapies have shown significant promise, with several immunomodulatory drugs being able to at least double the overall survival rates for patients with melanoma brain metastases^8^. These therapies may potentially be improved by radiation^37^ and they may have decreased adverse effects (e.g. decreased neurotoxicity)^38^. **

## **4. Immunotherapy in Metastatic Melanoma Tumors**

Current FDA approved treatment for melanoma are summarized in **Table 2**. Three of the most important targeted therapies used in the clinic are vemurafenib, trametinib, dabrafenib and some of their combinations, which are FDA approved regimens for melanoma treatment acting by blocking BRAF with activatory mutations, such as V600E or V600K. Notable, the chemotherapy treatment is ineffective, with only 5-20% of patients having their tumor shrink, but with no improvement in overall survival, although it was the only approved drug to treat metastatic melanoma (**Table 2**).

The first immunotherapeutic to show effect against melanoma brain metastasis was high dose interleukin 2 (hdIL-2). Melanoma patients with CNS involvement require higher doses of IL-2, which is challenging due to adverse events such as neurotoxicities and the need for hydration^39^. Recently, several immunomodulatory drugs were approved for melanoma treatment, with a recent study showing that the checkpoint blockade immunotherapy can double survival rates for patients with melanoma brain metastases^8^. Patients receiving these immunomodulatory drugs showed a mean survival of ~12.5 months compared to ~5.2 months for those not receiving immunotherapy, with a 4-year survival of ~28% versus only ~11%^8,40^.

**Figure 1:**
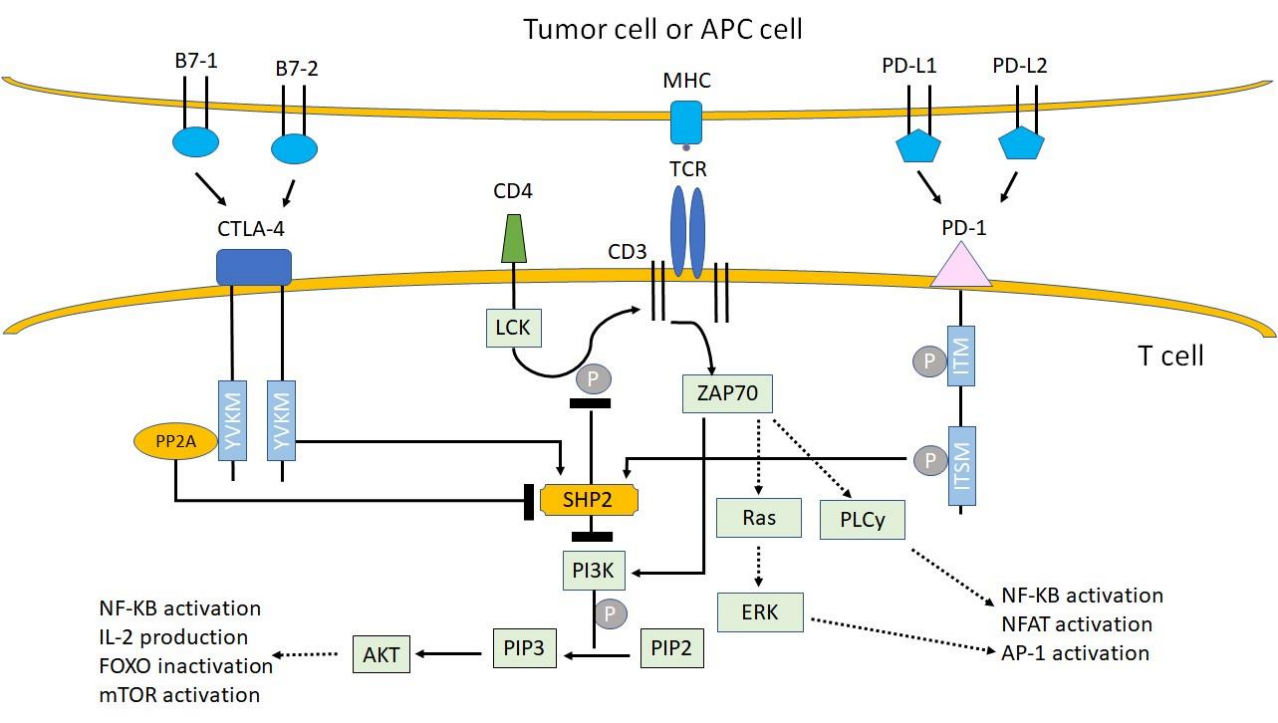
PD-1 and CTLA-4 checkpoint pathways. This figure presents the ligand-receptor interaction between tumor/APC cells and T cells, and activation of the PI3K-Akt, Ras-ERK and PLCy pathways inside the T cells.

Research studies have demonstrated the CD4 and CD8 are required for limitation or prevention of brain metastases, with an important role assigned to the regulatory T cells (Treg)^41^. The most important molecules as immune checkpoints are the programmed cell death protein 1 (PD-1) and its ligand (PD-L1) and the cytotoxic T lymphocyte-associated protein 4 (CTLA-4). PD-1 is found on the T cells and its interaction with PD-L1 expressed on the cancer cells surface, causes apoptosis of cytotoxic T lymphocytes, while preventing apoptosis of Treg cells^42^. In addition, CTLA-4 is a co-stimulatory protein which interacts with receptors on T lymphocytes, inhibiting effector T cells.**Both pathways are significant modulators of immune-tumor interaction (**Figure 1**) and targeting them focused significant energy in the past several years, with notable successes^38^.

Stimulation of T cells in the periphery with immunomodulators have also benefic effects against CNS tumors. A recent study has shown that pembrolizumab-induced PD-1 inhibition results in 20-30% responses in CNS, in patients with melanoma of non-small lung cancer CNS metastases. Moreover, combined regimen of nivolumab and ipilimumab, which acts by both inhibiting PD-1 and CTLA-4 has notable 55% CNS response in melanoma brain metastases patients^38^. Additionally, radiation therapy (e.g. SRS) is known to sensitize melanoma brain metastases to the action of checkpoint inhibitors, such as ipilimumab^43^. It is important to point out that, currently, there are several clinical trials underway for melanoma brain metastasis. A summary of the most important clinical trials is presented in **Table 3**. Immunomodulatory drugs, such as PD-1/PD-L1 or CTLA-4 inhibitors, have a great therapeutic potential in metastatic melanoma, including melanoma brain metastases. Noteworthy, only a small percentage of the patients are actually responding to these immunotherapies, with a high percentage of resistant cases. Thus, significant research has to be further performed in order to clearly define which patients respond to immune checkpoint inhibitors and how to sensitize the non-responders to these therapies.

**Table 3:** Clinical Trials in Melanoma Brain Metastasis Clinicaltrials.gov; SRS: stereotactic radiosurgery, WBRT: whole brain radiation.

IMMUNOTHERAPY	PHASE	TRIAL	NAME OF THE TRIAL
Pembrolizumab (anti-PD-1 antibody) plus Bevacizumab (anti-angiogenic)	II	NCT02681549	Pembrolizumab Plus Bevacizumab for Treatment of Brain Metastases in Metastatic Melanoma or Non-small Cell Lung Cancer
Pembrolizumab	II	NCT02886585	Pembrolizumab In Central Nervous System Metastases
Pembrolizumab	II	NCT02085070	MK-3475 in Melanoma and NSCLC Patients With Brain Metastases
Nivolumab (anti-PD-1 inhibitor)	II	NCT02621515	Nivolumab in Symptomatic Brain Metastases (CA209-322)
Fotemustine (alkylating agent) Fotemustine and Ipilimumab (anti-CTLA-4 inhibitor) Ipilimumab and Nivolumab	III	NCT02460068	A Study of Fotemustine (FTM) Vs FTM and Ipilimumab (IPI) or IPI and Nivolumab in Melanoma Brain Metastasis (NIBIT-M2)
Nivolumab vs. Nivolumab with Ipilimumab	II	NCT02374242	Anti-PD 1 Brain Collaboration for Patients With Melanoma Brain Metastases (ABC)
Nivolumab plus Ipilimumab followed by Nivolumab monotherapy	II	NCT02320058	A Study to Evaluate Safety and Effectiveness in Patients With Melanoma That Has Spread to the Brain Treated With Nivolumab in Combination With Ipilimumab Followed by Nivolumab by Itself (CheckMate204)
TARGETED THERAPY	PHASE	TRIAL	NAME OF THE TRIAL
Dabrafenib (BRAF inhibitor) plus Trametinib (MEK1/2 inhibitor)	II	NCT01978236	Dabrafenib/Trametinib, BRAF or BRAF AND MEK Pre-op With BRAF and MEK Post-op, Phase IIB, Melanoma With Brain Mets, Biomarkers and Metabolites
Buparlisib (pan-PI3K inhibitor)	II	NCT02452294	Buparlisib in Melanoma Patients Suffering From Brain Metastases (BUMPER)
Abemaciclib (CDK4/6 inhibitor)	II	NCT02308020	A Phase 2 Study of Abemaciclib in Patients With Brain Metastases Secondary to Hormone Receptor Positive Breast Cancer, Non-small Cell Lung Cancer, or Melanoma
WP1066 (STAT3 pathway inhibitor)	I	NCT01904123	A Phase I Trial of WP1066 in Patients With Recurrent Malignant Glioma and Brain Metastasis From Melanoma
Dabrafenib (BRAF inhibitor) plus Trametinib (MEK inhibitor)	II	NCT02039947	Study to Evaluate Treatment of Dabrafenib Plus Trametinib in Subjects With BRAF Mutation-Positive Melanoma That Has Metastasized to the Brain
Vemurafenib (BRAF inhibitor) plus Cobimetinib (MEK1/2 inhibitor)	II	NCT02537600	Vemurafenib and Cobimetinib Combination in BRAF Mutated Melanoma With Brain Metastasis (CONVERCE)
RADIATION PLUS SYSTEMIC THERAPY	PHASE	TRIAL	NAME OF THE TRIAL
Dabrafenib (BRAF inhibitor) plus SRS	II	NCT01721603	A Phase 2 Prospective Trial of Dabrafenib With Stereotactic Radiosurgery in BRAFV600E Melanoma Brain Metastases
Nivolumab (anti-PD1 antibody) plus SRS	Pilot	NCT02716948	Stereotactic Radiosurgery and Nivolumab in Treating Patients With Newly Diagnosed Melanoma Metastases in the Brain or Spine
Pembrolizumab (anti-PD1 antibody) plus SRS	Pilot	NCT02858869	Pembrolizumab and Stereotactic Radiosurgery for Melanoma or Non-Small Cell Lung Cancer Brain Metastases
Ipililumab (anti-CTLA-4 antibody) plus SRS	II	NCT02097732	Ipilimumab Induction in Patients With Melanoma Brain Metastases Receiving Stereotactic Radiosurgery
Ipilimumab (anti-CTLA-4 antibody) plus WBRT	II	NCT01703507	Phase I Study of Ipilimumab Combined With Whole Brain Radiation Therapy or Radiosurgery for Melanoma
Ipilimumab (anti-CTLA4 antibody) plus WBRT	II	NCT02115139	GEM STUDY: Radiation and Ipilimumab in Patients With Melanoma and Brain Metastases

## **5. Challenges and Limitations**

Treatment of melanoma brain metastases with surgery and/or radiation therapy results in a very low median overall survival and there are important complications and morbidity associated with these treatments, with a prominent cognitive decline (see **Table 1**). For example, WBRT can result in radiation toxicity, headaches, nausea, vomiting, bone marrow suppression, skin reactions, fatigue, while SRS is usually associated with neurocognitive decline, brain swelling, fatigue, skin problems, local hair loss, nausea, vomiting, headaches (**Table 1**). Focal treatments such as SRS and surgery are limited to the treatment of the area of interest, which may result if tumor relapse from other not treated sites which were under the limit of detection of our imaging methods^25^.

Resistance to radiation, chemotherapy, targeted treatments and recently developed immunotherapies is one of the major challenges in treating melanoma, melanoma brain metastases and other types of malignancies. For example, in the case of immunotherapy, a significant number of patients do not respond to existing immunotherapy treatments, and the exact causes are under investigation^38^. Brain metastases are generally resistant to cancer immunotherapy. An extensive understanding of these mechanisms and causes of resistance for brain metastases is required in order to overcome this resistance. One limitation to these investigations are the current methods used to investigate the tumor and in situ tumor microenvironment of the brain, which provide limited information of a heterogeneous tissue, spatially and dynamically, in time^27^. Another limitation is the lack of preclinical models which can mimic with high accuracy human brain metastases and that can recapitulate all the steps of brain metastases development^12^. As some research group suggest, the development of intravital microscopy technologies for high resolution imaging of brain metastases can be an important step forward^27^.

The majority of patients with melanoma brain metastases will receive some form of radiation therapy. Thus, it is important to investigate how radiotherapy interferes with targeted and immunotherapy. Although initially the radiation treatment was believed to be immunosuppressive, recent studies showed that it can actually serve a booster of the anti-tumor immune response, by increasing the availability of antigens available after radiation-induced necrosis and other mechanisms. Noteworthy, the combination of radiation with immunomodulatory drugs is more efficient than the use of each drug alone^38^.

It is now imperative to detect better biomarkers within the CNS which can guide the therapeutic strategy and can predict the response to therapy, in particular immunotherapy. For example, some studies show that higher density of CD3 and CD8 tumor-associated lymphocytes is a good prognostic factors correlated with increased survival^38^. Additionally, some treated patients with brain metastases may need control of their symptoms with steroids, which can make immunotherapy ineffective^38^. In conclusion, there are many challenges and limitations to overcome in order to better investigate, understand, develop effective therapies and significantly treat melanoma brain metastases.

## **6. Conclusion**

Melanoma patients with metastatic brain tumors have very poor prognosis. However, recent therapeutic strategies, such as the use of immunomodulatory drugs, are now emerging, with several compounds already approved by the FDA and other ones in clinical trials. Immunotherapy has already revolutionized the treatment of melanoma and other malignancies, with very effective results and low adverse events for some of the treated patients. However, many of the patients are resistant to immunotherapy and it is imperative to find out the exact mechanisms and how the tumor can be rendered sensitive. The preliminary studies in melanoma brain metastasis show significant promise and require additional investigation. In conclusion, immunotherapy and immunomodulatory drugs bring a great promise as new tools for melanoma treatment in particular and for the treatment of other types of malignancies in general.**

## KEY POINTS

◊ Some immunomodulatory drugs are able to at least double the overall survival rates for patients with melanoma brain metastases

◊ Immunotherapy, including the immunomodulatory drugs, bring a great promise as new tools for melanoma treatment in particular, and for the treatment of other types of malignancies in general.

## References

[1] Langley Robert R, Fidler Isaiah J (2013). The Biology of Brain Metastasis. Clinical Chemistry.

[2] Patchell Roy A (2003). The management of brain metastases. Cancer Treatment Reviews.

[3] Johnson John D., Young Byron (1996). Demographics of Brain Metastasis. Neurosurgery Clinics of North America.

[4] Lassman Andrew B, DeAngelis Lisa M (2003). Brain metastases. Neurologic Clinics.

[5] Farber S. Harrison, Tsvankin Vadim, Narloch Jessica L., Kim Grace J., Salama April K. S., Vlahovic Gordana, Blackwell Kimberly L., Kirkpatrick John P., Fecci Peter E. (2016). Embracing rejection: Immunologic trends in brain metastasis. OncoImmunology.

[6] Cruz-Muñoz William, Kerbel Robert S. (2011). Preclinical approaches to study the biology and treatment of brain metastases. Seminars in Cancer Biology.

[7] Jindal Vishal, Gupta Sorab (2018). Expected Paradigm Shift in Brain Metastases Therapy—Immune Checkpoint Inhibitors. Molecular Neurobiology.

[8] Iorgulescu J. Bryan, Harary Maya, Zogg Cheryl K., Ligon Keith L., Reardon David A., Hodi F. Stephen, Aizer Ayal A., Smith Timothy R. (2018). Improved Risk-Adjusted Survival for Melanoma Brain Metastases in the Era of Checkpoint Blockade Immunotherapies: Results from a National Cohort. Cancer Immunology Research.

[9] What is Cancer Immunotherapy. American Cancer Society. Accessed in June 2019:.

[10] Soare Georgiana R., Soare Costin A. (2019). Immunotherapy for Breast Cancer: First FDA Approved Regimen. Discoveries.

[11] Chang Liisa, Chang Minna, Chang Hanna M., Chang Fuju (2017). Microsatellite Instability. Applied Immunohistochemistry & Molecular Morphology.

[12] Puhalla S., Elmquist W., Freyer D., Kleinberg L., Adkins C., Lockman P., McGregor J., Muldoon L., Nesbit G., Peereboom D., Smith Q., Walker S., Neuwelt E. (2015). Unsanctifying the sanctuary: challenges and opportunities with brain metastases. Neuro-Oncology.

[13] Davies Michael A., Liu Ping, McIntyre Susan, Kim Kevin B., Papadopoulos Nicholas, Hwu Wen-Jen, Hwu Patrick, Bedikian Agop (2010). Prognostic factors for survival in melanoma patients with brain metastases. Cancer.

[14] Quail Daniela F, Joyce Johanna A (2013). Microenvironmental regulation of tumor progression and metastasis. Nature Medicine.

[15] Chen Qing, Boire Adrienne, Jin Xin, Valiente Manuel, Er Ekrem Emrah, Lopez-Soto Alejandro, S. Jacob Leni, Patwa Ruzeen, Shah Hardik, Xu Ke, Cross Justin R., Massagué Joan (2016). Carcinoma–astrocyte gap junctions promote brain metastasis by cGAMP transfer. Nature.

[16] Aspelund Aleksanteri, Antila Salli, Proulx Steven T., Karlsen Tine Veronica, Karaman Sinem, Detmar Michael, Wiig Helge, Alitalo Kari (2015). A dural lymphatic vascular system that drains brain interstitial fluid and macromolecules. The Journal of Experimental Medicine.

[17] Louveau Antoine, Smirnov Igor, Keyes Timothy J., Eccles Jacob D., Rouhani Sherin J., Peske J. David, Derecki Noel C., Castle David, Mandell James W., Lee Kevin S., Harris Tajie H., Kipnis Jonathan (2015). Structural and functional features of central nervous system lymphatic vessels. Nature.

[18] Louveau Antoine, Harris Tajie H., Kipnis Jonathan (2015). Revisiting the Mechanisms of CNS Immune Privilege. Trends in Immunology.

[19] Weiss Nicolas, Miller Florence, Cazaubon Sylvie, Couraud Pierre-Olivier (2009). The blood-brain barrier in brain homeostasis and neurological diseases. Biochimica et Biophysica Acta (BBA) - Biomembranes.

[20] Berghoff Anna S, Fuchs Elisabeth, Ricken Gerda, Mlecnik Bernhard, Bindea Gabriela, Spanberger Thomas, Hackl Monika, Widhalm Georg, Dieckmann Karin, Prayer Daniela, Bilocq Amelie, Heinzl Harald, Zielinski Christoph, Bartsch Rupert, Birner Peter, Galon Jerome, Preusser Matthias (2015). Density of tumor-infiltrating lymphocytes correlates with extent of brain edema and overall survival time in patients with brain metastases. OncoImmunology.

[21] Quail Daniela F., Joyce Johanna A. (2017). The Microenvironmental Landscape of Brain Tumors. Cancer Cell.

[22] Osswald Matthias, Jung Erik, Sahm Felix, Solecki Gergely, Venkataramani Varun, Blaes Jonas, Weil Sophie, Horstmann Heinz, Wiestler Benedikt, Syed Mustafa, Huang Lulu, Ratliff Miriam, Karimian Jazi Kianush, Kurz Felix T., Schmenger Torsten, Lemke Dieter, Gömmel Miriam, Pauli Martin, Liao Yunxiang, Häring Peter, Pusch Stefan, Herl Verena, Steinhäuser Christian, Krunic Damir, Jarahian Mostafa, Miletic Hrvoje, Berghoff Anna S., Griesbeck Oliver, Kalamakis Georgios, Garaschuk Olga, Preusser Matthias, Weiss Samuel, Liu Haikun, Heiland Sabine, Platten Michael, Huber Peter E., Kuner Thomas, von Deimling Andreas, Wick Wolfgang, Winkler Frank (2015). Brain tumour cells interconnect to a functional and resistant network. Nature.

[23] Lin Xuling, DeAngelis Lisa M. (2015). Treatment of Brain Metastases. Journal of Clinical Oncology.

[24] Staudt M, Lasithiotakis K, Leiter U, Meier F, Eigentler T, Bamberg M, Tatagiba M, Brossart P, Garbe C (2010). Determinants of survival in patients with brain metastases from cutaneous melanoma. British Journal of Cancer.

[25] Cohen Justine V., Kluger Harriet M. (2016). Systemic Immunotherapy for the Treatment of Brain Metastases. Frontiers in Oncology.

[26] Hottinger Andreas F., Pacheco Patricia, Stupp Roger (2016). Tumor treating fields: a novel treatment modality and its use in brain tumors. Neuro-Oncology.

[27] Owyong Mark, Hosseini-Nassab Niloufar, Efe Gizem, Honkala Alexander, van den Bijgaart Renske J.E., Plaks Vicki, Smith Bryan Ronain (2017). Cancer Immunotherapy Getting Brainy: Visualizing the Distinctive CNS Metastatic Niche to Illuminate Therapeutic Resistance. Drug Resistance Updates.

[28] Nowak-Sadzikowska Jadwiga, Walasek Tomasz, Jakubowicz Jerzy, Blecharz Paweł, Reinfuss Marian (2016). Current treatment options of brain metastases and outcomes in patients with malignant melanoma. Reports of Practical Oncology & Radiotherapy.

[29] Yamamoto M, Serizawa T, Shuto T, Akabane A, Higuchi Y, Kawagishi J (2014). Stereotactic radiosurgery for patients with multiple brain metastases (JLGK0901): a multi-institutional prospective observational study. Lancet Oncol.

[30] Glitza Oliva Isabella, Tawbi Hussein, Davies Michael A. (2017). Melanoma Brain Metastases: Current Areas of Investigation and Future Directions.. The Cancer Journal.

[31] Chapman Paul B., Hauschild Axel, Robert Caroline, Haanen John B., Ascierto Paolo, Larkin James, Dummer Reinhard, Garbe Claus, Testori Alessandro, Maio Michele, Hogg David, Lorigan Paul, Lebbe Celeste, Jouary Thomas, Schadendorf Dirk, Ribas Antoni, O'Day Steven J., Sosman Jeffrey A., Kirkwood John M., Eggermont Alexander M.M., Dreno Brigitte, Nolop Keith, Li Jiang, Nelson Betty, Hou Jeannie, Lee Richard J., Flaherty Keith T., McArthur Grant A. (2011). Improved Survival with Vemurafenib in Melanoma with BRAF V600E Mutation. New England Journal of Medicine.

[32] Hauschild Axel, Grob Jean-Jacques, Demidov Lev V, Jouary Thomas, Gutzmer Ralf, Millward Michael, Rutkowski Piotr, Blank Christian U, Miller Wilson H, Kaempgen Eckhart, Martín-Algarra Salvador, Karaszewska Boguslawa, Mauch Cornelia, Chiarion-Sileni Vanna, Martin Anne-Marie, Swann Suzanne, Haney Patricia, Mirakhur Beloo, Guckert Mary E, Goodman Vicki, Chapman Paul B (2012). Dabrafenib in BRAF-mutated metastatic melanoma: a multicentre, open-label, phase 3 randomised controlled trial. The Lancet.

[33] FDA Approved Drugs. Aim at Melanoma Foundation. Accessed in June 2019.

[34] Wolchok Jedd D Immunotherapy for Melanoma: How is Immunotherapy Changing the Outlook for Patients with Melanoma?. Cancer Research Institute. Accessed in June 2019.

[35] Chen Guo, Davies Michael A. (2012). Emerging insights into the molecular biology of brain metastases. Biochemical Pharmacology.

[36] McQuade Jennifer, Davies Michael A (2015). Converting biology into clinical benefit: lessons learned from BRAF inhibitors. Melanoma Management.

[37] Postow Michael A., Callahan Margaret K., Barker Christopher A., Yamada Yoshiya, Yuan Jianda, Kitano Shigehisa, Mu Zhenyu, Rasalan Teresa, Adamow Matthew, Ritter Erika, Sedrak Christine, Jungbluth Achim A., Chua Ramon, Yang Arvin S., Roman Ruth-Ann, Rosner Samuel, Benson Brenna, Allison James P., Lesokhin Alexander M., Gnjatic Sacha, Wolchok Jedd D. (2012). Immunologic Correlates of the Abscopal Effect in a Patient with Melanoma. New England Journal of Medicine.

[38] Kamath Suneel D., Kumthekar Priya U. (2018). Immune Checkpoint Inhibitors for the Treatment of Central Nervous System (CNS) Metastatic Disease. Frontiers in Oncology.

[39] Atkins Michael B., Lotze Michael T., Dutcher Janice P., Fisher Richard I., Weiss Geoffrey, Margolin Kim, Abrams Jeff, Sznol Mario, Parkinson David, Hawkins Michael, Paradise Carolyn, Kunkel Lori, Rosenberg Steven A. (1999). High-Dose Recombinant Interleukin 2 Therapy for Patients With Metastatic Melanoma: Analysis of 270 Patients Treated Between 1985 and 1993. Journal of Clinical Oncology.

[40] Immunotherapy doubles survival rates for patients with melanoma brain metastases. Brigham and Women's Hospital. Accessed in June 2019.

[41] Shevach Ethan M. (2011). Biological Functions of Regulatory T Cells. Advances in Immunology.

[42] Francisco Loise M., Sage Peter T., Sharpe Arlene H. (2010). The PD-1 pathway in tolerance and autoimmunity. Immunological Reviews.

[43] Knisely Jonathan P. S., Yu James B., Flanigan Jaclyn, Sznol Mario, Kluger Harriet M., Chiang Veronica L. S. (2012). Radiosurgery for melanoma brain metastases in the ipilimumab era and the possibility of longer survival. Journal of Neurosurgery.

